# Case Report: A comparative evaluation of anterior open bite orthodontic treatment outcomes and stability of results in monozygotic twins

**DOI:** 10.3389/froh.2026.1789325

**Published:** 2026-03-25

**Authors:** Olimpia Bunta, Mihaela Pastrav, Dana Festila

**Affiliations:** Orthodontics Department, Faculty of Dental Medicine, Iuliu Hatieganu University of Medicine and Pharmacy, Cluj-Napoca, Romania

**Keywords:** anterior open bite, highpull headgear, monozygotic twins, orthodontic treatment, temporary anchorage device, tongue thrust

## Abstract

Anterior open bite treatment represents an orthodontic challenge, mainly due to its’ intricate etiology. This report presents the evolution of the orthopedic and orthodontic treatment of two female monozygotic twins, aged 10. Both twins presented dental and skeletal anterior open bite, tongue thrust, oral breathing, narrow maxilla with protrusion and fake spaces, dental and skeletal class 2, a hyperdivergent growth pattern and cervical vertebrae maturation stage 3. The therapeutic approach for both twins included an ENT examination, orthopedic treatment by means of High Pull Head Gear, trans palatal bar with an acrylic button at the level of the palatal loop, myofunctional exercises addressing the tongue thrust and the tonicity of the perioral muscles, a Roth 0.022 inch fixed preadjusted appliance and a tongue crib. Treatment results were different in the two patients, as for one of the twins tongue thrust was eliminated, enabling orthopedic and orthodontic correction of the open bite and presenting no signs of relapse at follow-up appointment, while for the other twin tongue thrust persisted, therefor the orthodontic correction of the open bite was only partial and presented with tendency toward relapse at follow-up appointment. Although the orthodontic diagnosis and therapeutic approaches were similar for both patients, the clinical outcomes were different mainly due to patients’ compliance in the management of the tongue thrust and possibly, in the wearing protocol of the High Pull Head Gear.

## 1 Introduction

Open bite malocclusion is a vertical discrepancy characterized by the lack of contact between the upper and lower teeth (1–3). There are two types of open bite malocclusion based on the location of the teeth involved: anterior open bite, when the absence of vertical contact located between upper and lower teeth extends from canine to canine and posterior open bite, when the anomaly involves lack of vertical contact between premolars and molars. Anterior open bite is more common than posterior open bite, with a prevalence of 16.52% in children and adolescents aged 2–16 years (3, 4). In deciduous and early mixed dentition, the prevalence of anterior open bite is higher than in late mixed dentition, 11% vs. 4.2–6.2% respectively. In addition, in the permanent dentition, the prevalence reported by the literature is 2.5–8.7% (5).

Sassouni divided the open bite malocclusion into two categories: skeletal and dental open bite (6, 7). Skeletal open bite is associated with excessive vertical development of the dentoalveolar complex in the posterior maxillary region, increased lower anterior facial height and short posterior facial height, increased gonial angle and mandibular plane angle (8–13). Patients with skeletal open bite exhibit a hyperdivergent facial type, a clockwise rotation of the mandible, a retrognathic mandibular position and/or a counterclockwise rotation of the maxilla (14). Dental open bite is related to reduced vertical height of the dentoalveolar complex in the anterior region, normal craniofacial pattern and balanced lower anterior facial height (8, 14).

The etiology of the open bite malocclusion is complex, with both genetic and epigenetic factors contributing to the development of this anomaly (8). Among genetic factors linked to open bite are genes FGRR2, TCOF1, FAM83H, ENAM, AMGX, PAX5, PTPN11 and SOS1 (14–16). Studies have shown that genetic expression can be altered under the influence of epigenetic factors, impacting growth, the development of maxillary and mandibular bones and, also, tooth development (8).

This paper presents the comparative evaluation of the open bite treatment and stability of the results obtained orthodontically in a pair of monozygotic twins. Both hereditary and functional factors (tongue thrusting habit overlapped) were suspected as etiological causes of the malocclusion. In theory, similar treatment outcomes would be expected when following the same treatment protocol for twins with similar initial diagnosis, but different results raise questions regarding the cause of the malocclusion, its’ impact on the treatment outcomes and stability of the results.

## 2 Case description

**Figure 1:**
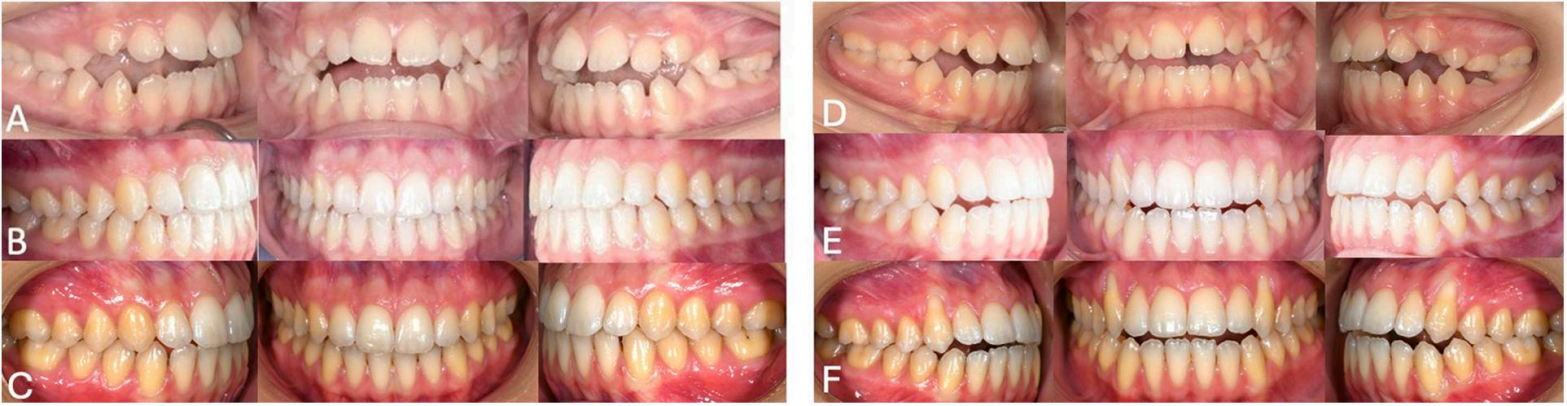
Intraoral evolution: **(A)** Twin A - before treatment; **(B)** Twin A – end of treatment; **(C)** Twia A - follow-up; **(D)** Twin B – before treatment; **(E)** Twin B – after debonding; **(F)** Twin B - **(D)** Twin B – follow-up.

**Figure 2:**
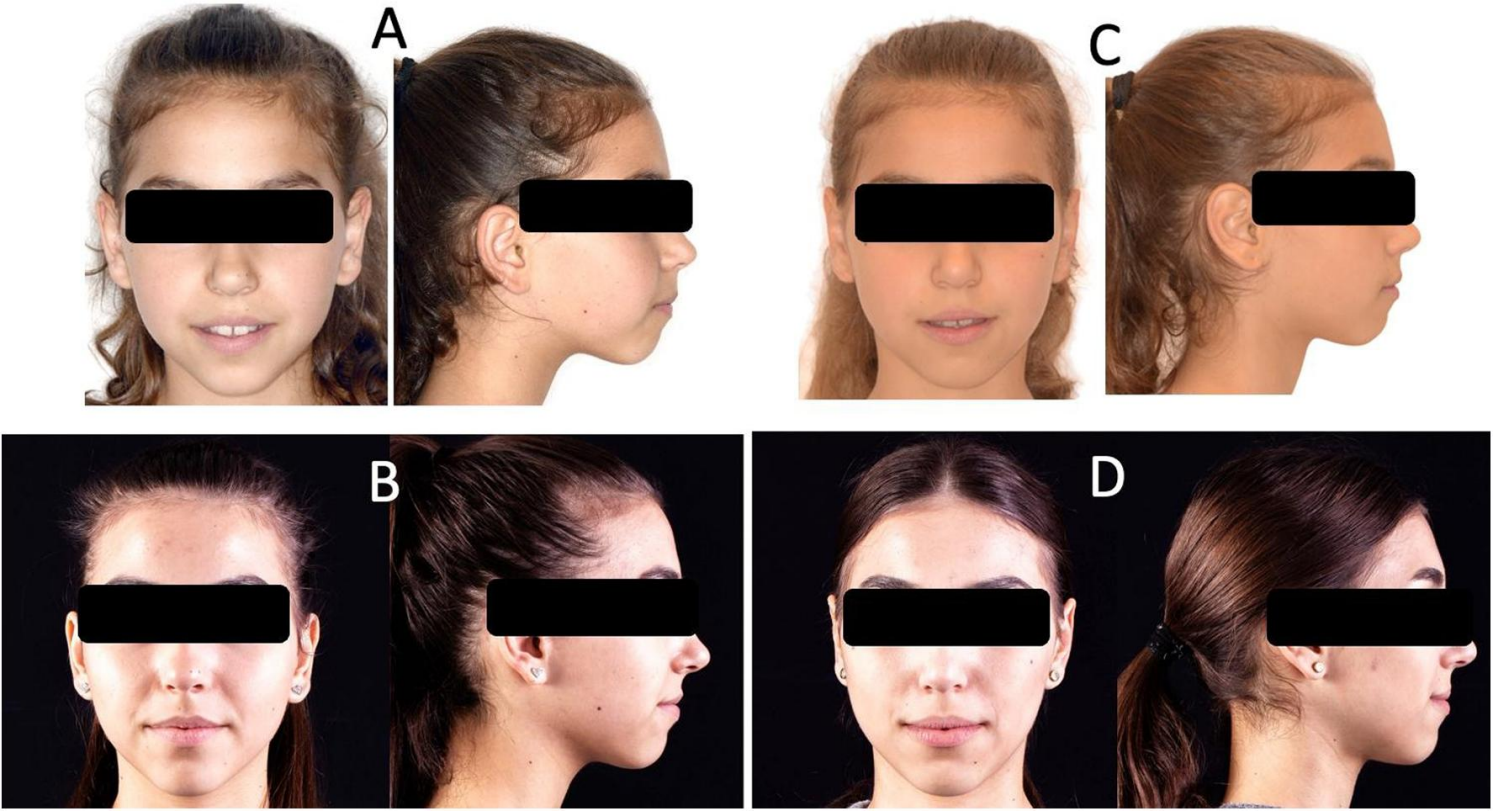
Facial evolution: **(A)** Twin A - before treatment; **(B)** Twin A - after treatment; **(C)** Twin B - before treatment; **(D)** Twin B - after debonding.

Two monozygotic twin sisters aged 10, presented in the Orthodontics Department of the Iuliu Hatieganu University of Medicine and Pharmacy, Cluj-Napoca, Romania, with a primary complaint of an impaired esthetic function, especially in speech. Clinical, photographical, orthodontic cast models and radiological evaluation (panoramic and lateral radiographs) of the patients revealed a mixed dentition, tongue thrust (atypical swallowing), oral breathing, dental and skeletal open bite, narrow maxilla with protrusion and fake spacing, dental and skeletal Class 2, an enlarged lower facial third, a hyperdivergent growth pattern and cervical vertebrae maturation (CVM) stage 3 (Figures 1A–D, 2A–C). As both patients presented skeletal and dental-alveolar discrepancies in the vertical and sagittal planes, associated with functional disorders, differential diagnosis could be made with temporomandibular and postural disorders, and syndromic and systemic conditions. Based on the clinical and radiological evaluation, no modifications were detected at the level of the temporo-mandibular joint (TMJ) and based on the pediatric medical records, both patients presented no systemic conditions. Perioral soft tissues presented as contracted in both patients in the closed mouth position.

Considering the orthodontic diagnosis, the treatment objectives were to: eliminate the tongue thrust parafunction; achieve a clockwise rotation of the maxilla and a counterclockwise rotation of the mandible; obtain an appropriate anterior overbite in the vertical plane; achieve Class 1 relations in the sagittal plane; achieve transversal coordination of the two dental arches; obtain stable results.

A written consent for patients’ treatment and patients’ information and images to be published was obtained from the parents prior to commencement of the treatment, in accordance with ethical guidelines given by the Ethics Committee of the “Iuliu Hatieganu” University of Medicine and Pharmacy Cluj-Napoca, Romania no. 20/20.01.2014.

**Figure 3:**
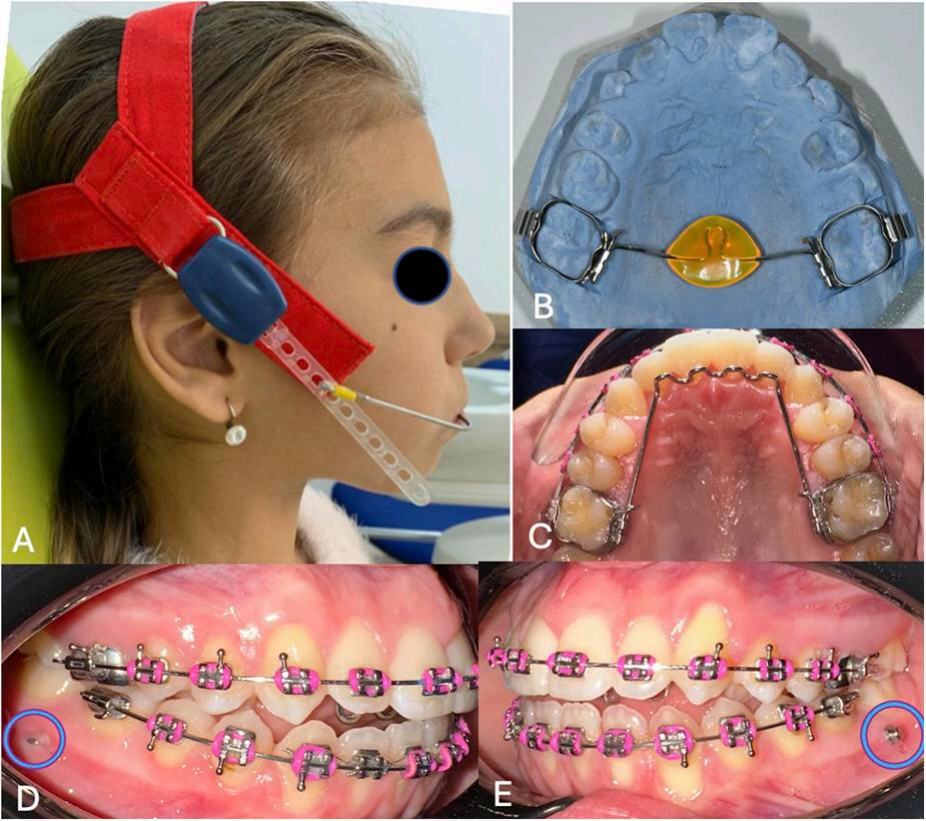
Appliances and accessories used during the treatment of the two patients: **(A)** Twin A- high pull head gear; **(B)** Twin A – trans palatal bar with acrylic button; **(C)** Twin B – fixed tongue crib; **(D, E)** – Twin B - temporary anchorage devices in the lower ach.

Initial treatment was started equally on both twins, at the same time, and began with an otorhinolaryngology (ENT) examination, which ruled out the presence of possible obstructions and the absence of adenoid vegetations. Given the patients’ diagnosis, tendency toward vertical growth and hyperdivergency, orthopedic treatment was initiated with the application of a High Pull Head Gear device (Figure 3A) which had the purpose of intruding the upper molars and favor a clockwise maxillary rotation. The forces applied were 250 g/side. The patients were indicated to wear the device 12–14 h/day in order to obtain a skeletal effect. To counteract the vestibular tipping effect at the level of the molars, a trans palatal bar was attached. Additionally, the TPA was constructed with an acrylic button at the level of the palatal loop (Figure 3B), in order to reorientate the tongue towards the palate and use its’ force to add an additional intrusion effect on the upper molars.

At the beginning of the orthopedic treatment, myofunctional therapy was also indicated in both twins in order to correct the position of the tongue at rest and during swallowing, and also to help with toning the perioral musculature. Both patients were instructed to perform the following exercises in order to re-educate the function of the tongue: “tongue spot” - involves locating the palatal spot behind the maxillary incisors, keeping the tongue on the spot for 10 s and repeating the exercise 10 times a day; positioning the tongue at the level of the acrylic button of the TPA, keeping this position for 10 s and repeating the exercise 10 times a day; “tongue click” – involves placing the tongue against the palate and making a clicking sound; “4S (spot, salivate, squeeze, swallow) exercise” – involves placing the tongue on the spot behind the maxillary incisors, keep the position while salivating, followed by the act of squeezing the spot and ultimately swallowing the saliva. For toning the perioral musculature, especially the lips, the following exercises were indicated: lip closure using an ice cream stick in-between the lips for 5–10 s and repeating the exercise 5–10 times/ day; “the ballooning exercise” – involves blowing into a balloon; straw drinking - involves using a straw in order to consume liquids. The indication of performing the above mentioned myofunctional exercises was kept throughout the entire treatment, orthopedic and orthodontic.

After the orthopedic phase, treatment continued with the application of a fixed preadjusted appliance, Low Profile, American Orthodontics (AO, Sheboygan, Wisconsin, USA) with a Roth.022-inch slot prescription on the upper lateral segments (segmental canine - second premolar fixed appliance), and the application of an extended TPA with bands and molar tubes at the level of the first molars, with a lingual crib (Figure 3C) to maintain the results obtained from wearing the HPGH appliance and also to coordinate the tongue into a correct position, this being one of the factors that led to the patients’ open bite. Application of brackets only in the lateral areas of the maxillary arch aimed to prevent protrusion of the frontal group and correct the sagittal relations at the level of the canines and molars. The objectives of this treatment phase were achieved in approximately 10 months of treatment, followed by the application of brackets at the level of the anterior teeth, in order to continue with the alignment and leveling of the dental arch, correct the occlusal plane and close the anterior open bite. A fixed metal appliance with Roth 0.22 prescription, from the same producer, was applied in the mandibular arch, where the classical sequence of arch wires was followed in order to align and level the teeth, correct the occlusal plane, as well as closing the interdental spaces.

The anterior open bite was further approached by intercuspation elastics in a box configuration, 6 oz, size 3/16 inch, with the indication to wear at least 20 h/day, and extrusion bands on 0.016 × 0.016 inch stainless steel arch wires.

### 2.1 Treatment progress

A different duration of the orthopedic phase of treatment was observed, even with declared patient compliance and in the same wearing conditions of the HPHG. For Twin A, this phase of treatment lasted 10 months, while for Twin B, it lasted 16 months.

**Figure 4:**
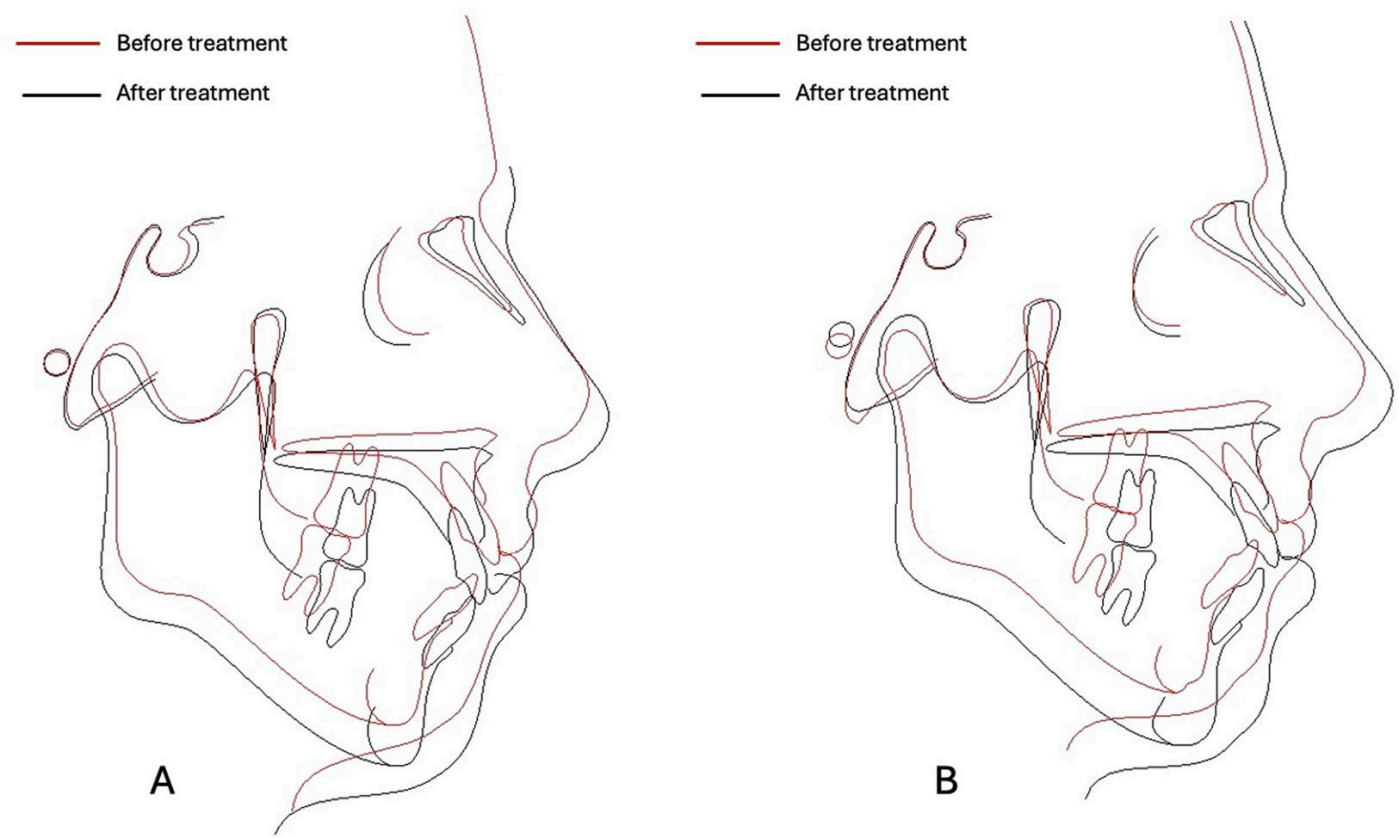
Superimposition of cephalometric evaluation: **(A)** Twin A - before and after treatment; **(B)** Twin A – before and after debonding.

From the comparative analysis of the seriated lateral x-rays (before and after debonding) some representative values have been selected and are illustrated in Table 1. As can be observed from Table 1 and the cephalometric superimpositions (Figure 4), for Twin A a counterclockwise mandibular rotation and a clockwise rotation of the maxilla occurred, alongside with both a relative extrusion and, partially, an absolute extrusion of the maxillary and mandibular incisors, which resulted in normal vertical occlusal relationships (Figures 1B, 4A). The total amount of open bite reduction for Twin A was of 4 mm, measured from the incisal edge of the central maxillary incisors to the incisal edge of the central mandibular incisors, the patient presenting a 1.5 mm of vertical overbite at the moment of debonding. For Twin B, the anterior open bite was corrected only partially, as no considerable skeletal modifications were observed, no significant relative extrusion and only a certain degree of absolute extrusion of the maxillary and mandibular incisors was achieved (Figures 1E, 4B). The total amount of open bite reduction for Twin B was of 2.5 mm, measured from the incisal edge of the central maxillary incisors to the incisal edge of the central mandibular incisors, the patient presenting 0 mm of vertical overbite at the moment of debonding.

**Table 1:** Teleradiographical measurements for both twins before and after orthodontic treatment.

MEASUREMENT	Twin A		Twin B	
	Before treatment	After treatment	Before treatment	After treatment
Sn-GoGn (Angle of anterior cranial base to mandibular plane)	37.7°	36.6°	38°	39.1°
SNA	85.3°	82.2°	84.1°	82.6°
SNB	78.2°	78.9°	78°	78.1°
ANB	7.1°	3.3°	6.2°	4.5°
NL-NSL (Anterior cranial base - palate)	3°	5°	4°	4°
ar-Go (Ramus length)	35 mm	40 mm	32 mm	36 mm
Go-Me (Mandibular length)	60 mm	69 mm	63 mm	72 mm
Sn-OcP (Anterior cranial base to occlusal plane)	21.3°	24.4°	20.6°	17.9°
Ii (Interincisal angle)	108°	126.9°	108.2°	117.3°
Max1-NA (Angle of axis of 1u to N-A)	24.9°	20.2°	26.7°	27.6°
Max1-SN (Angle of axis of 1u to S-N)	110.2°	102.4°	110.9°	110.1°
Mand1-NB (Angle of axis of 1l to N-B)	40°	29.7°	38.9°	30.6°
1u-NA (Distance of labial outline of 1u to N-A)	4 mm	5 mm	5 mm	5 mm
1l-NB (Distance of labial outline of 1l to N-b)	9mm	6 mm	7 mm	7 mm

Twin A was able to control and correct the tongue position during orthodontic treatment, thus achieving incisor overbite and maintaining the position over time without interposing the tongue at the dental level. On the other hand, for Twin B, tongue control was not obtained, therefore the lack of minimum coverage ratios at the incisors’ level and tongue persistence and interposition despite the presence of the lingual shield. Oral breathing diagnosed initially in both twins, resulted as being adaptative to the anterior open bite, as for Twin A this parafunction was completely absent at the end of the treatment, and for Twin B the oral beathing transformed into a mixed breathing, most probably due to the open bite partial reduction.

For obtaining a counterclockwise mandibular rotation, control of the dental eruption in the posterior areas and molar intrusion were necessary, therefore the use of the HPHG at the moment of the growth peak. For Twin B, additional bilateral buccal intra-alveolar temporary anchorage devices (TADs) were used in the mandibular arch (Figures 3D, E), to attempt the intrusion of the molars. TADs failure after two different attempts, made the patient and parents request orthodontic treatment finalization, even though the anterior open bite remained partially uncorrected. The patient and parents were informed about the need for orthognathic surgery in the future and the possible periodontal consequences of this decision.

At the end of the treatment, in the case of Twin A we have achieved the first five initial treatment objectives. In the case of Twin B, out of the initial treatment objectives we have managed to successfully achieve sagittal Class 1 and transversal coordination of the two arches. Tongue thrust was still present at the moment of debonding, consequently only partial vertical improvement being achieved.

Total treatment duration was of 40 months for Twin A, respectively, 46 months for Twin B.

Retention of the results was obtained by means of an Essex retainer in the maxillary arch and a fixed canine to canine retainer in the mandibular arch - Twin A, respectively a fixed canine to canine retainer in the maxillary arch and a fixed first bicuspid to first bicuspid retainer in the mandibular arch – Twin B.

### 2.2 Follow-up

Follow-up photographs are presented in Figures 1C, F. The follow-up orthodontic consult was performed 22 months after orthodontic treatment finalization for Twin A, respectively, 16 months after orthodontic appliance debonding in Twin B.

In Twin A, the results of the orthodontic treatment are stable, as tongue thrust has been corrected. For Twin B, the tongue thrust is still present, with an obvious tendency of the open bite towards relapse (0.6 mm relapse measured between the incisal edges of the maxillary and mandibular incisors). As a consequence of the decision to terminate orthodontic treatment before treatment finalization, lateral guidances in Twin B are improper, thus leading to gingival recession at the level of both maxillary canines.

## 3 Discussion

Environmental factors such as tongue malfunction, thumb sucking habit, prolonged pacifier use, disproportionate neuromuscular growth can alter the mechanical forces applied on the teeth, contributing to the development of open bite malocclusion (8, 14, 17–26). Tongue interposition between dental arches during swallowing or at rest is the most frequent environmental factor related to anterior open bite, but there is a debate in the literature whether tongue thrusting is the cause or the effect of this malocclusion (17).

In this context, the study of twins are useful for analyzing the effects of genetic and epigenetic factors on phenotypic expression (27). There are two types of twins: monozygotic or identical twins and dizygotic or fraternal twins (28). Monozygotic twins are the result of the fertilization of a single egg that is divided into two identical embryos, sharing identical genetic material (27–30). Therefore, the resulting embryos manifest identical phenotypic characteristics (29). Dizygotic twins originate from a separate zygote, therefore they share half of their genetic information, and the phenotypic characteristics are as similar as are those of siblings (27, 28, 30). Although monozygotic twins have identical genetic material, they can exhibit phenotypic differences as a result of the interaction between genetic and epigenetic factors in their life (29, 30). Thus, oral habits such as tongue thrusting, thumb sucking, mouth breathing and imbalance of neuromuscular forces influence dentofacial development, producing phenotypic variations among monozygotic twins (29).

Multiple studies and case reports in the literature have presented successful correction of open bite malocclusion and long term stability of the results in the context of tongue thrust elimination (31, 32). Stabel correction by means of first premolar extractions and High Pull Head Gear was reported even in young adults (33). Myofunctional therapy combined with a fixed palatal crib has been proven as efficient in growing patients (34). Other approaches found as successful in the literature involve second premolar extractions and a fixed palatal crib (35). Analyzing the literature, it emerges that patient compliance in deconditioning the tongue thrust parafunction is the key factor to treatment success.

Therefore, the main strength of this report is to highlight the influence and impact of parafunctional disorders in the etiology, the treatment and the stability of the results in anterior open bite cases. Patient compliance stands out as one of the most important aspects of the therapeutic approach, if not the most important, as extraoral forces and myofunctional exercises are necessary in order to treat anterior open bite of parafunctional etiology.

A limitation of this report is represented by the fact that no actual genetic testing to prove monozygosity was performed, the affirmation being based on the initial information provided by the mother when taking the patients’ medical history and subsequent confirmation of this information by the twins’ pediatrician.

## 4 Conclusion

Based on the analysis of the two twins presented in this report, the conclusion that the tongue thrust was the cause of the initial orthodontic malocclusion, can be drawn. Eliminating this parafunctional behavior of the tongue enabled dental-alveolar and skeletal orthopedic and orthodontic modifications, therefore obtaining a stable correction of the anterior open bite for one of the twins. Persistence of the tongue thrust, even in the context of combined orthopedic, myofunctional and orthodontic approaches, attributable to the lack of patient compliance, lead only to a partial unstable correction of the initial anterior open bite.

The comparative results presented in this report highlight the causal effect of tongue thrust in the etiology, treatment and stability of results in anterior open bite cases, and the importance of patient compliance in terms of therapeutic approaches.

## Data Availability

The original contributions presented in the study are included in the article/Supplementary Material, further inquiries can be directed to the corresponding authors.
